# Costs of Stroke and Incidence of First Diagnosis of Atrial Fibrillation at Time of Stroke. Neurology Ward Hospital Poznań, Poland 2018

**DOI:** 10.3390/healthcare9080999

**Published:** 2021-08-05

**Authors:** Tomasz Zaprutko, Jolanta Florczak-Wyspiańska, Dorota Kopciuch, Anna Paczkowska, Piotr Ratajczak, Jolanta Dorszewska, Elżbieta Nowakowska, Krzysztof Kus

**Affiliations:** 1 Department of Pharmacoeconomics and Social Pharmacy, Poznan University of Medical Sciences, 7 Rokietnicka St, 60-806 Poznan, Poland; dorota.koligat@gmail.com (D.K.); aniapaczkowska@ump.edu.pl (A.P.); p_ratajczak@ump.edu.pl (P.R.); kkus@ump.edu.pl (K.K.); 2 Department of Neurology, Poznan University of Medical Sciences, 49 Przybyszewskiego St, 60-355 Poznan, Poland; jolaflorczak@ump.edu.pl (J.F.-W.); dorszewskaj@yahoo.com (J.D.); 3 Department of Toxicology and Pharmacology, University of Zielona Góra, 28 Zyty St, 65-046 Zielona Góra, Poland; farmakoekonomika@ump.edu.pl

**Keywords:** stroke, costs, atrial fibrillation, inpatient care, academic centre

## Abstract

Stroke is a major cause of morbidity in industrialized countries, representing 8% of total deaths across Europe in 2017. It is also a very costly disorder, frequently caused by atrial fibrillation. We aimed to calculate the cost of stroke hospitalization in 2018 in Poznań (Poland). We also intended to present patients with the first AF diagnosis at the time of stroke. The study was conducted from January 2019 to July 2020. Data were obtained from hospital records and from the hospital accounting department. Out of 164 patients included in the study, 41 had AF and in 18 cases AF was first diagnosed at the time of stroke. The cost of hospitalization in Poznań was EUR 139,257.21 ($\bar{x}=$ EUR 849.13). Among those with concomitant AF, the general cost of inpatient care was EUR 33,859.18 ($\bar{x}=$ EUR 825.83). Considering those who had AF first diagnosed during hospitalization the cost was EUR 16,248.97 ($\bar{x}=$ EUR 906.24). Stroke is associated with high costs of inpatient care, which turned out to be higher among those with AF first diagnosed at the time of stroke. The number of patients who used oral anticoagulants at the time of admission was relatively low. The most frequently used NOAC was dabigatran.

## 1. Introduction

Stroke is one of the most significant worldwide healthcare problems with substantial health, economic and social consequences hampering the ability of survivors to perform everyday activities [1,2,3]. It contributed to 0.4 million deaths in 32 European countries in 2017 [3]. The global lifetime risk of stroke is about 25% from the age of 25 [4]. In the European Union (EU), Switzerland, Iceland and Norway approximately 1.1 million new stroke events occur annually [5]. In the United States (US), stroke prevalence is similar to European countries and varies between 1.9% and 4.3% among those older than 20 [6]. Importantly, the incidence of stroke significantly increases with age and doubles every decade after the age of 55 [6]. It makes stroke the third or even the second cause of death worldwide [1,2,4,7,8,9].

Stroke prevalence, the increasing number of affected patients and rapidly aging societies [5,7,10,11,12] contribute to the fact that stroke is characterized by a significant economic burden [1,5,11,13]. In the study published in 2020 by Puciarelli et al., the direct cost for stroke in Europe was calculated at EUR 16 billion, whereas in the US it was USD 23.6 billion [6]. By 2035 these costs are expected to increase to USD 66 billion [6]. Just in the UK, however, societal and annual costs were estimated at £26 billion, including costs covered by the National Health Service (13%), Personal Social Services (20%), unpaid care (61%), and productivity loss (6%) [4].

Apart from common risk factors (e.g., diabetes, hypertension and smoking) for stroke [8], atrial fibrillation (AF) is also a major cause of ischemic stroke and is associated with about one third of all strokes [10,14]. If AF, however, is diagnosed early and anticoagulant treatment is introduced simultaneously, 64% of AF-related strokes might be prevented [15,16]. Despite a decrease in stroke mortality in western countries [8] and progress in stroke prevention, there is still space for improvements which may contribute to further reduction of stroke-related costs [4]. One of the most interesting AF detection and stroke prevention methods is mobile health technology based on mobile and wireless devices such as smartphones with a dedicated application [10,15,17]. Although the European Society of Cardiology (ESC) recommends opportunistic AF screening among patients aged ≥ 65 years, this is not routinely performed [10,18,19].

Nevertheless, up to 25% patients have AF diagnosed only after the stroke event [14,20]. Apart from health consequences, it increases the economic and public health burden of stroke [6,14]. Hence, to save patients from stroke related disability and premature death, and to reduce costs, effective stroke management is urgently needed [7].

The aim of our study was to calculate cost of stroke hospitalization at a stroke unit in 2018 in Poznań (Poland). We also intended to present the percentage of patients with first AF diagnosis at the time of stroke. In turn, this percentage will make it possible to evaluate the potential benefits of common AF screening.

## 2. Material and Methods

The retrospective study was conducted from January 2019 to July 2020, and was suddenly stopped by the COVID-19 pandemic. It analysed the costs of inpatient care of stroke in 2018 at the Neurology Ward of the Heliodor Święcicki Hospital of Poznań University of Medical Sciences (Poland). Poznań is the capital of the Greater Poland region, which is inhabited by almost 3.5 million of citizens. It covers an area of about 30,000 km^2^, and it represents almost 10% of Poland’s territory, making its area is comparable to Belgium, for instance.

The inclusion criteria were as follows: stroke diagnosed on the basis of the International Classification of Diseases, Tenth Revision (ICD-10) and adult patients (>18 years of age). Patients were excluded from the analysis, however, if they were transferred to another ward or hospital. The study conforms with the Personal Data Protection Act and each hospital record was considered anonymously.

Data were obtained from hospital records and from the hospital accounting department. At first, we collated a list of all patients hospitalized in 2018 at the Neurology Ward in Poznań. After that we identified those patients who received the first AF diagnosis at the time of stroke.

The cost of inpatient care of stroke in Poznań is presented as a total cost, including hospital stay, pharmacotherapy, non-pharmacological care and diagnostic tests. It results from the funding of hospitalization in Poznań, where individual components of cost per day are covered regardless of the patients’ sex. The general cost was calculated by adding values corresponding to each patient.

Amounts in PLN were converted to EUR at the average EUR exchange rate in 2018 (study concerns patients hospitalized in 2018) published by the National Bank of Poland (EUR 1 = PLN 4.2623). Monetary values presented in the study have been calculated by converting monetary units into the common European currency and then rounded.

## 3. Statistics

The data were shown as mean values ± SEM (plus the median and minimum/maximum dates). The data distribution pattern was not normal (unlike the Gaussian function). The data were evaluated using non-parametric tests (Kruskal–Wallis, Mann–Whitney U) for unpaired data. Correlations between each group were calculated using the Dunn and/or Dunnett’s test. Statistical significance was adopted at *p* < 0.05.

## 4. Results

In 2018 there were 166 patients hospitalized at the stroke unit in Poznań (female; *n* = 65; 39.16%). According to the study criteria, 2 hospital records (male) were excluded and 164 hospital records were ultimately investigated (65 female and 99 male).

Altogether, there were 41 patients with concomitant AF. However, 18 patients (43.90%) were first diagnosed with AF at the time of stroke, of which 11 (61.11%) were female.

There were 23 deaths (14.02%) in the analysed group, of which 13 (56.52%) were female. In that group 9 patients had AF, and 6 of them had AF first diagnosed during hospitalization at the stroke unit. The structure of the study group followed by observed diagnoses is presented in Table 1.

The group of analysed patients was hospitalized for 2903 days, of which 1209 days were attributable to females. Patients of both sexes generated a total direct cost of EUR 139,257.21 ($\bar{x}=$ EUR 849.13). The cost of the female and male groups was EUR 55,384.18 ($\bar{x}=$ EUR 852.06) and EUR 83,873.03 ($\bar{x}=$ EUR 847.20), respectively.

In the group of patients with concomitant AF, the general cost of inpatient care was EUR 33,859.18 ($\bar{x}=$ EUR 825.83). The costs for females and males were EUR 17,720.71 ($\bar{x}=$ EUR 843.84) and EUR 16,138.47 ($\bar{x}=$ EUR 806.92), respectively.

Considering those who had AF first diagnosed during hospitalization, the total cost of their inpatient care was EUR 16,248.97 ($\bar{x}=$ EUR 906.24). In the case of females and males, the costs were EUR 10996.18 ($\bar{x}=$ EUR 999.65) and EUR 5316.14 ($\bar{x}=$ EUR 759.45), respectively.

The analysis of anticoagulants used at the time of hospital admission revealed that 14 (8.54%) patients used these medicines, of which 64.29% used novel oral anticoagulants (NOACs). A detailed breakdown of the used NOACs is presented in Table 2.

## 5. Discussion

In this study, our principal finding was that the total cost of inpatient care at the stroke unit in Poznań constituted EUR 139,257.21 ($\bar{x}=$ EUR 849.13). AF was first diagnosed at the time of stroke for 18 patients (10.98%). Although patients with previously unknown AF were hospitalized for shorter periods, the cost of their inpatient care (EUR 16,248.97; $\bar{x}=$ EUR 906.24) was higher as compared to the whole study group. The higher costs could mainly result from diagnostic tests performed and medicines used by those patients. However, the shorter hospitalizations (hospitalizations lasting 1 or 2 days) might be explained by the number of deaths in the group of patients with AF first diagnosed at the stroke unit. It confirms the significance of early AF detection and immediate introduction of anticoagulant treatment, contributing to its cost-effectiveness [6,7,13,21,22].

The cost of cardiovascular diseases, including stroke, was globally about USD 863 billion, with predictions rising to USD 1.044 billion by 2030 [12]. Almost 50% of these costs relate to the US. In Poland, these costs ranged from EUR 8.2 billion to over EUR 9.6 billion for the 2015–2017 period [12]. In 2017 stroke-related healthcare costs accounted for €27 billion (45%), representing 1.7% of health expenditure in 32 European countries [3]. According to the Burden of Stroke in Europe report [23] the healthcare costs of stroke in Poland accounted for EUR 561 million. Costs of inpatient care remain the largest component of the total direct medical costs associated with stroke [13]. Nevertheless, values related to the mean cost per patient may significantly differ between countries [7]. In our study it was $\bar{x}=$ EUR 849.13. In Japan and in the USA the mean daily costs per patient were USD 209 and USD 551, respectively [7]. However, Wei et al. [24] estimated that, on average, the overall cost of hospitalization in China was about USD 1600. These discrepancies may result from differences in the length of hospital stay (LoHS) observed in several studies. In our study it was almost 18 days. Yoneda et al. pointed out that in Japan it was 33 days, and 8 days in the USA [7]. In China LoHS was 20 days [24]. In the Netherlands, however, on average it was almost 9 days [9]. Nevertheless, Epstein et al. [25] revealed that the total LoHS was almost 16 days in the Netherlands and 14.7 days in Poland.

The costs of stroke also arise from the age of patients. In South Korea, for instance, the age distribution of stroke patients is moving toward younger patients, thus the economic burden is set to increase both in terms of direct and indirect costs [4,26]. Kang et al. [26] revealed that the cost of stroke is almost doubled if the stroke onset is at the age 45 compared to onset at 75, and 10% higher at the age of 65 as compared to 75. In our study, the mean age of patients was 74 years old, which is in line with the Polish Stroke Registry ($\bar{x}=$ 75) [1]. Hence, patients in Poland were older on average than those in Japan ($\bar{x}=$ 70), USA ($\bar{x}=$ 71), China ($\bar{x}=$ 72) and the Netherlands ($\bar{x}=$ 71) [7,9,13].

If AF is diagnosed early and oral anticoagulation therapy is prescribed, 64% of AF-related strokes might be prevented and thus costs could be reduced [15,16,20,27]. Today, one of the most interesting AF detection methods is screening via mobile devices (e.g., smartphones) [10,17]. This non-invasive and cost-effective method has turned out to be feasible at community pharmacies. It made it possible to detect 1.33% and 1.5% of patients with previously unknown AF in Poland and Australia, respectively [10,17,28]. Furthermore, Aronsson et al. [29] revealed that screening for asymptomatic AF would result in about 270 fewer strokes among 75-year-old Swedes.

The importance of AF screening and early detection of this cardiac arrhythmia is in line with the aim and results of our study. We revealed that 10.98% of patients received their first AF diagnosis at the time of stroke. However, this figure is lower than in other studies. Borowsky et al. [14] found that in the USA, 18% of patients were first diagnosed with AF at the time of stroke, which was modestly lower than the 24% observed in Sweden. In Finland, AF was first diagnosed in 22% of patients hospitalized for an ischemic stroke [16]. In Japan, however, 29% of patients had their first diagnosis of AF at hospital admission or during stroke hospitalization [30].

It is also interesting to analyse the impact of patients’ sex on stroke prevalence and costs. Despite the importance of this point, it seems that these facets have not been well characterized and thus need more analysis [31]. In our study, there were more males (*n* = 99 vs. 65 females). In every category (Table 1) the average cost was higher for females. However, this may vary across different studies because of the study participants’ age. According to Reeves et al. [32], in older age groups, and due to their longer life expectancy and much higher incidence at older ages, women have more stroke events than men. This has been confirmed by Kolominsky-Rabas et al. [33] who observed slightly higher costs and rate of stroke incidence among women in Germany. However, a report from Australia indicates, in total, a slightly greater incidence of stroke among males, mainly resulting from the higher incidence expectancy among males aged 55–84. If patients were younger than 55 or aged >85, the trend was reversed. However, further studies concerning sex differences in stroke-related healthcare costs are needed, e.g., to highlight areas of inequity or generate possible ways to reduce costs, issues which are relevant for policy makers and health system planning [31].

An analysis of hospital records revealed that in our study 8.54% of patients used anticoagulants before admission to hospital, and 64.29% of this cohort used NOACs. Among those who had AF, 31.71% were using anticoagulants at the time of admission, but in the group of patients who received the first AF diagnosis at the time of stroke, this figure was 16.67% (all of whom used NOACs). The Burden of Stroke in Europe report revealed that in Poland, 39.8% of stroke patients with AF were using oral anticoagulants pre-stroke [23]. The authors of that report also indicated that, out of 95% of AF patients eligible for oral anticoagulants, only 39% are given prescriptions. This is despite 44,276 deaths attributable to stroke annually.

In Spain, more than 40% of patients are not treated according to European clinical guidelines for patients with AF, which recommend anticoagulation therapy [21]. In the US, Borowsky et al. [14] pointed out that among patients who were discharged from a stroke unit, 75% were on anticoagulants or had a care plan to start such pharmacotherapy within four weeks of hospital discharge. This is in line with ESC guidelines suggesting that most patients with a stroke risk are viable candidates for anticoagulation therapy [18].

Warfarin remains effective in prevention strategies for patients with AF and reduces the risk of stroke by 64% [8,34]. However, the risk of bleeding, variability in metabolism, or delayed onset of action are the drawbacks of warfarin therapy [34]. Currently, NOACs offer relative safety and efficacy compared to warfarin [34]. These benefits have been demonstrated in several clinical trials [21]. This is in line with our study, where among those who were on anticoagulants during admission the majority used NOACs. The most frequently used NOAC during admission was dabigatran, used by 50% of patients on anticoagulants and 66.67% of patients with AF first diagnosed at the time of stroke. In our study, dabigatran was followed by apixaban. This is reflected by comparisons of NOACs. Rutherford et al. [35] and Villines et al. [22] concluded that dabigatran and apixaban were associated with a significantly lower risk of bleeding compared with rivaroxaban. Nevertheless, these substances were not different in terms of effectiveness [36]. Rutherford et al. [35] also revealed that both rivaroxaban and dabigatran were associated with a higher risk of gastro-intestinal bleeding compared with apixaban. In turn, Staerk et al. [36] concluded that dabigatran was associated with a lower intracranial bleeding risk compared with rivaroxaban and apixaban. Nonetheless, several studies confirm the importance of the common use of NOACs for the prevention of stroke and related disorders [21,22,34].

## 6. Limitations

We are aware that it would be valuable to present data from a few centres. Nevertheless, our study might be considered an important source of information, for example, due to the fact that analyses from East European countries (such as Poland) have previously been indicated as needed in the field [5]. It could be interesting to present the costs of pharmacotherapy, laboratory tests and hospital stays separately. However, the structure of hospital records and funding of inpatient care in Poland meant that we were only able to present the total cost of hospitalization. Besides, it would be valuable to present costs at purchasing power parity (PPP), which reduces the scope of between-country variations. This might be the aim of further multicentre analyses, which we planned to conduct, but were not possible due to the COVID-19 pandemic. It would also be interesting to present data on the exact pharmacotherapy used during inpatient care and to compare the share of NOACs in the study group. Another limitation of the study is that we have not investigated post-stroke cognition and systemic inflammation [37], another promising area for future research. Due to the limitations of our study, the results should be extended onto a larger sample and the conclusions should be updated.

## 7. Conclusions

Stroke is associated with high costs of inpatient care. Patients with AF first diagnosed at the time of stroke constituted almost 11% of the study group and generated higher hospitalization costs than the whole study group. AF, a leading cause of stroke, is preventable and should be effectively detected using new technologies such as, e.g., smartphone-based screening to further reduce the costs of hospitalization. The number of patients who were using oral anticoagulants at the time of admission was relatively low.

## Figures and Tables

**Table 1:** The structure of the study group and related diagnoses.

Patients		$\mathrm{Age}\;\mathrm{in}\;\mathrm{Years}\;(\bar{x})\;\pm \;\mathrm{SEM}$	$\mathrm{Cos}t\;\mathrm{in}\;\mathrm{EUR}\;(\bar{x})\;\pm \;\mathrm{SEM}$	Hospitalization Length—Days			Diagnosis	
				$(\bar{x})\;\pm \;\mathrm{SEM}$	Maximum	Minimum	Cerebral Infarction	Intracerebral Hemorrhage
Total (*n* = 164)		73.96 ± 1.07	849.13 ± 36.28	17.70 ± 1.59	199	1 ^#^	134	30
Female (*n* = 65)		76.80 ± 1.86 *NS*	852.06 ± 80.58 *NS*	18.60 ± 3.27 *NS*	199	2	52	13
Male (*n* = 99)		72.09 ^^^ ± 1.27 (*p =* 0.0316)	847.20 ± 29.01 *NS*	17.11 ± 1.55 *NS*	105	1 ^#^	82	17
AF	Total (*n* = 41)	79.81 * ± 1.75 (*p* = 0.0126)	826.84 ± 83.14 *NS*	15.93 ± 2.4 *NS*	105	1 ^#^	38	3
	Female (*n* = 21)	84.95 ** ± 1.51 (*p =* 0.0182)	843.84 ± 156.06 *NS*	13.67 ± 1.50 *NS*	29	3	20	1
	Male (*n* = 20)	74.40 ^^^ ± 2.79 (*p =* 0.0017)	806.92 ± 53.64 *NS*	18.30 ± 4.86 *NS*	105	1 ^#^	18	2
AF de novo ^&^	Total (*n* = 18)	79.83 ± 2.73 *NS*	906.24 ± 178.40 *NS*	13.78 ± 1.89 *NS*	28	1 ^#^	18	-
	Female (*n* = 11)	84.64 ± 2.25 *NS*	999.65 ± 286.84 *NS*	14.18 ± 2.11 *NS*	24	3 ^#^	11	-
	Male (*n* = 7)	72.29 ^^^ ± 5.04 (*p =* 0.0221)	759.45 ± 102.52 *NS*	13.14 ± 3.77 *NS*	28	1 ^#^	7	-

^#^ Hospitalization finished due to the patient’s death. ^&^ AF first diagnosed during hospitalization at stroke unit. SEM—Standard error of the means. * Statistically significant difference vs TOTAL. ** Statistically significant difference vs. TOTAL female. ^^^ Statistically significant difference vs. female.

**Table 2:** Anticoagulants used at the time of admission to the hospital.

Anticoagulants Used at the Time of Admission to the Hospital		Total	AF ^^^	AF De Novo ^&^
Altogether		14	13	3
NOACs	Rivaroxaban	1	-	-
	Apixaban	1	1	1
	Dabigatran	7	7	2

^^^ Including those with previously known AF and with AF first diagnosed during hospitalization at stroke unit. ^&^ AF first diagnosed during hospitalization at stroke unit.
